# How does organizational AI adoption affect employees’ job crafting behaviors? An approach-avoidance perspective

**DOI:** 10.3389/fpsyg.2025.1690238

**Published:** 2026-01-09

**Authors:** Qi Liu, Qing Tian, Xin Li, Hongjuan Tan

**Affiliations:** 1 School of Business, Macau University of Science and Technology, Macao, Macao SAR, China; 2 School of Economics and Management, Civil Aviation Flight University of China, Guanghan, China; 3 School of Management, Guangzhou Xinhua University, Guangzhou, Guangdong, China

**Keywords:** organizational AI adoption, AI-supported autonomy, AI anxiety, approach-avoidance job crafting, AI knowledge sharing

## Abstract

**Introduction:**

Artificial intelligence (AI) technology has significantly changed human work. Increasingly, organizations are promoting the integration of AI into employees’ work processes. While existing research has explored AI applications in the workplace, relatively little attention has been devoted to understanding how organizational AI adoption influences employees’ motivational reactions and the subsequent impacts. Drawing on approach-avoidance motivational theory, this research explores the fundamental mechanisms through which organizational AI adoption influences employees’ approach and avoidance job crafting behaviors.

**Methods:**

A three-wave survey was conducted among 650 employees from five enterprises that actively utilize AI tools in China, yielding 487 valid responses. Data were analyzed using SPSS 21.0 and Mplus 7.0 to test the proposed hypotheses.

**Results:**

The findings suggest that organizational AI adoption influences employees’ approach or avoidance motivation, which in turn shapes their job crafting behaviors. Specifically, AI-supported autonomy mediates the positive relationship between organizational AI adoption and approach job crafting. Conversely, AI anxiety mediates the positive relationship between organizational AI adoption and avoidance job crafting. Furthermore, AI knowledge sharing moderates the effects of organizational AI adoption on motivational reactions and the indirect effects of organizational AI adoption on approach and avoidance job crafting, respectively.

**Discussion:**

This study introduces the approach-avoidance perspective, providing a valuable framework for understanding employees’ motivational responses and job crafting behaviors following the organizational AI adoption, thereby expanding the application scope of the approach-avoidance motivational theory. Organizations should leverage the role of AI in knowledge sharing to enhance employees’ approach motivation and reduce their avoidance motivation. Future research may focus on developing an AI-specific job crafting construct and exploring additional antecedents of AI-related crafting behaviors.

## 1 Introduction

Artificial intelligence (AI) has emerged as a transformative force in organizational environments, with the potential to enhance productivity and reduce operational costs (Duan et al., 2019; Huo et al., 2022). Enterprises across different industries are increasingly integrating AI into their operation. For instance, in service firms, AI chatbots/virtual voice assistants can be designed to improve customer’ support (Ren, 2021). Similarly, in the financial industry, AI-based algorithms and new risk management systems are growingly appearing as guides for investment decisions (Guo and Polak, 2023). The application of AI not only enhances employees’ productivity but also helps them achieve their work goals more efficiently (Sowa and Przegalinska, 2020). These developments have contributed to a growing body of research in recent years. Although many studies primarily focused on the individual AI acceptance (e.g., Prentice et al., 2020; Wei and Li, 2022), employees’ AI adoption (e.g., Pelau et al., 2021), and AI awareness (e.g., Kong et al., 2021; He G. et al., 2023; Liu et al., 2025), the organizational adoption of AI has received less scholarly attention (Lin et al., 2024). Notably, workforce operations are undergoing significant modifications as a result of AI implementation (Zhao et al., 2025), research on the impact of organizational AI adoption—a significant external factor—on employees’ work behaviors and outcomes remains limited. Therefore, given that the interaction between employees and AI in the organizational environment is an important topic in AI-related research in the field of management (Makarius et al., 2020), it is of great significance to investigate how the organizational AI adoption affects the psychology and behavior of employees.

The emergence of AI is profoundly altering and will continue to reshape the design, demands, and boundaries of traditional jobs, as well as the competencies required in the coming years (Wang et al., 2017; Tong et al., 2021). Faced with this trend, both scholars and practitioners are confronted with a fundamental question: how can employees proactively adapt rather than passively respond to this transformation (Bailey and Barley, 2020; Parker and Grote, 2022). Employees may engage in job crafting—actively adjusting their tasks and roles—to adapt to organizational changes amid a complex and ambiguous work environment (Budhwar et al., 2022; Petrou et al., 2015). Job crafting encompasses task crafting, relational crafting, and cognitive crafting (Wrzesniewski and Dutton, 2001). Tims et al. (2012) integrated job crafting into the job demands-resources model, while Lichtenthaler and Fischbach (2019) examined it through regulatory focus theory (Higgins, 1997), distinguishing between promotion and prevention-focused crafting.

Additionally, Bruning and Campion (2018) applied approach-avoidance motivation theory (Elliot, 2006) to categorize job crafting as either approach or avoidance-oriented. Although extensive studies have explored the effects of job crafting on employee outcomes, including their sense of meaning and identity (Wrzesniewski et al., 2013), job exhaustion (Petrou et al., 2015), adaptive performance (Wang et al., 2017), and perceived employability (Petrou and Xanthopoulou, 2021), the factors and mechanisms that influence job crafting have been given relatively little scholarly attention. Prior investigations into the antecedents of job crafting have primarily focused on individual characteristics, such as proactive personalities (Bakker et al., 2012); environmental influences, such as transformational leadership (Wang et al., 2017) and human resource strategies (Kooij et al., 2022); and job characteristics, such as performance pressure (Kang et al., 2023) and job complexity (Tian et al., 2022). However, the impact of the integration of new technologies into organizations (e.g., organizational AI adoption) on employees’ job crafting has not been comprehensively explored. This situation prompts critical inquiry regarding the relationship between organizational AI adoption and employees’ job crafting behavior.

Besides, most current research in the management field on AI adoption or AI usage has predominantly employed the three types of theoretical frameworks: the Transactional Model of Stress (TMS) theory (Folkman and Lazarus, 1988), the Job Demand-Resource (JD-R) model (Demerouti et al., 2001), and the Conservation of Resources (COR) theory (Hobfoll et al., 2018).

The Transactional Model of Stress (TMS) theory outlines the cognitive processes and subsequent responses when facing specific stressors (Folkman and Lazarus, 1988). In research using the TMS theory in an AI context, the impact of digital technology on employees has been studied as work-related stressors. AI-related technologies could cause stress in employees if introduction of those new systems demands to use unfamiliar tasks and new jobs and skills (Park and Cho, 2022; Kang et al., 2023; Yin et al., 2024). The Job Demands-Resources (JD-R) model can be used to consider the employee’s resource level and work engagement by focusing on job demands and resources (Demerouti et al., 2001; Bakker et al., 2003). The model has been applied in the literature a plenty of times to study the effect of job factors on outcomes among workers (Song et al., 2024). A significant number of studies grounded in the JD-R theory conceptualize AI technology or employees’ AI awareness as a job demand, investigating its effects—either beneficial or detrimental—on performance and innovative behaviors through two underlying mechanisms: the strain pathway and the motivation pathway (Liang et al., 2022; He G. et al., 2023). Based on the Conservation of Resources (COR) theory (Hobfoll, 1989), individuals strive to prevent net resource loss by utilizing or investing resources they already possess or can access within their environment (Peng et al., 2022). The people under pressure are more likely to have insufficient available resources, which would result in the deteriorate work performance. In AI-related research based on COR theory, most studies takes resource conservation viewpoint as perception, and the pressure of technology, or increased AI awareness, is taken as the employee’s resource consumption, and then studies how the consumption leads to the employee’s negative work behaviors (Shen and Kuang, 2022; Choi, 2023; Teng et al., 2024).

When examining scholarly literature concerning AI’s effects on personnel, these three theoretical frameworks predominantly consider AI technology as a source of stress, a job demand, or a depletion of personal resources, and conduct studies by focusing on the potential negative impacts of AI technology on employees. There is a lack of an objective and neutral perspective that treats the use of AI technology as an objectively existing external factor and then analyzes how the adoption of new technologies, represented by AI, in the organizational environment affects employees’ motivational responses and ultimately influences their positive or negative work behaviors. To conclude, a significant gap remains in our understanding of how organizational AI adoption affects employees’ job crafting behaviors, particularly through the lens of motivational response mechanisms.

In summary, the purpose of this research is to address these gaps in existing knowledge by investigating the fundamental mechanisms and boundary conditions through which organizational AI adoption influences employees’ job crafting behavior, using the approach-avoidance motivational theory as a framework.

Based on the approach-avoidance motivational theory (Elliot, 1999; Elliot and Church, 1997; Elliot and Thrash, 2002; Elliot, 2006), this study attempts to gain a deeper understanding of the dual impact of organizational AI adoption on employees’ job crafting behaviors. Approach-avoidance motivational theory is a psychology-based perspective that has garnered the attention of researchers in both psychology and management. According to Elliot (2006), approach-avoidance motivation refers to the activation or direction of behavior toward positive stimuli, such as objects, events, or possibilities, which is known as approach motivation, while avoidance motivation involves directing behavior away from negative stimuli to prevent contact with them. The fundamental connotation of this theoretical framework is that the pursuit of pleasure and the avoidance of pain are the basic driving forces of human behavior (Elliot and Thrash, 2002; Ferris et al., 2011). By integrating this theoretical framework into the context of organizational AI adoption, this study aims to explore the motivational mechanisms that drive employees to approach or avoidance job crafting behaviors.

To further analyze the motivational reaction process of employees, we propose that when organizational AI adoption as a specific source of external context, it may activate either approach or avoidance motivational responses (i.e., AI-supported autonomy and AI anxiety), thereby generating approach-avoidance job crafting behaviors. Organizational AI adoption can stimulate employees’ approach motivation by enhancing their sense of AI-supported autonomy, thereby motivating proactive changes such as approach job crafting (Petrou et al., 2018; He G. et al., 2023).

As the work environment evolves with the integration of AI, various motivational mechanisms may emerge (Elliot, 2006; Ferris et al., 2011). To be specific, AI holds the promise of enhancing both the effectiveness and productivity of a wide range of tasks, encouraging humans to respond with a sense of control and accomplishment in interactions with AI systems. These emotions—especially the autonomy supported by AI—help build employees’ confidence and acceptance of AI technologies (Bai et al., 2025), motivating them to collaborate proactively with AI and engage in behaviors such as approach job crafting. On the other hand, organizational AI adoption may also activate employees’ avoidance motivation (i.e., AI anxiety), which may prompt employees to engage in avoidance job crafting behaviors. The adoption of artificial intelligence by organizations may also stimulate employees’ avoidance motivation (i.e., AI anxiety), which may prompt employees to engage in avoidance-oriented job crafting behaviors. Incorporating AI into work processes may evoke negative emotions such as fear, anxiety, and worry (Konuk et al., 2023), thereby generating avoidance responses. Specifically, AI-driven digitization reshapes work structure, increases job uncertainty, competitiveness, and technological adaptation challenges (Lu et al., 2024). The pressures may translate into AI anxiety, which reinforces avoidance motivation. As a result, employees might be reluctant to collaborate with AI and could engage in avoidance job crafting behaviors, such as reducing their work role and withdrawal.

Given that organizational AI adoption can impact employees’ job crafting behaviors, we examine why some employees amplify their approach. In contrast, others weaken their avoidance motivation in response to organizational AI adoption (Xu et al., 2021). To delineate the boundary conditions of this relationship, drawing on approach-avoidance motivational theory (Elliot, 2006), we conceptualize this divergence through the lens of achievement motives—fundamental orientations toward pursuing competence (approach) and evading incompetence (avoidance) (McClelland, 1985; Sommet et al., 2019). Specifically, we propose AI knowledge sharing as a key moderating variable. AI knowledge sharing activates achievement motives, amplifying approach motivation and strengthening the positive link between organizational AI adoption and AI-supported autonomy (e.g., proactive job crafting). In so doing, it also reduces avoidance motivations and buffers the adverse effects of AI anxiety, such as reducing work withdrawal. In this way, AI knowledge sharing is vital in determining whether employees embrace AI-driven opportunities or retreat from AI-induced threats, and consequently, what the outcomes of their job crafting will be.

This research makes several important theoretical contributions to the existing literature.

First, by analyzing how organizational AI adoption influences employees’ job crafting through approach-avoidance motivation, we deepen understanding of employee responses and contribute to AI research in organizational behavior. Secondly, we enrich the literature on job crafting by analyzing the specific motivational mechanisms that drive approach or avoidance job crafting in a working environment that collaborates with artificial intelligence. We elaborated in detail on how employees can reshape their work, whether by proactively embracing the job opportunities brought by AI (e.g., approach job crafting) or retreating from the anxiety triggered by AI (e.g., avoidance job crafting). Third, by integrating the approach-avoidance motivational theory (Elliot, 2006), this study presents an innovative theoretical framework that offers a promising approach to enhancing our comprehension of how employees respond positively or negatively to organizational AI adoption. It also expands the application of approach-avoidance motivational theory (Elliot, 2006) by examining the mediating roles of approach and avoidance motivation (i.e., AI-supported autonomy and AI anxiety) in organizational AI adoption and behavioral reactions. Fourth, we contribute to the research on knowledge management by revealing the moderating role of AI knowledge sharing in the relationship between organizational AI adoption and motivational responses. Specifically, we demonstrate how AI knowledge sharing can amplify positive employee motivational outcomes (e.g., AI-supported autonomy) while reducing negative motivations (e.g., AI anxiety), thereby leading to varied job crafting behaviors.

## 2 Theoretical background and research hypotheses

### 2.1 Theoretical background

To better understand these processes, we draw upon the approach-avoidance motivation framework—a well-established theoretical perspective frequently used to explain and anticipate motivational tendencies and behavioral responses (Elliot, 2006; Ferris et al., 2011; Venkatesh et al., 2003). According to Elliot (2006), this framework centers on “the energization of behavior toward positive stimuli (approach motivation) or away from negative stimuli (avoidance motivation).” It posits that human actions are driven by two fundamental and opposing systems: the pursuit of pleasure (approach) and the avoidance of pain (avoidance) (Elliot and Thrash, 2010; Ferris et al., 2011). Within this model (Elliot, 2006), approach motives are likely to stimulate positive behaviors and avoidance motives are likely to induce negative behaviors. Originating in psychological theory, the approach-avoidance framework has increasingly been adopted in organizational and management studies.

In organizational behavior, several studies applied the approach-avoidance motivational theory. Xu et al. (2021) examined how performance pressure affects employees’ in-role behaviors. Howard and Smith (2023) explored how employee regret and disappointment influence organizational citizenship and counterproductive behaviors through approach and avoidance motives. Impett et al. (2014) studied the emotional and relational outcomes of sacrificing for approach versus avoidance goals, considering both givers and recipients. Lu et al. (2022) found that hospitality employees facing customer mistreatment may adopt either avoidance or approach job crafting strategies, which differently impact job performance.

To sum up, we find that in the current research on organizational behavior based on approach-avoidance motivational theory, most studies examine how negative situations (e.g., mistreatment; performance pressure) or negative emotion (e.g., sacrificing; regret; disappointment) in the workplace affect employees’ behavior or performance through approach-avoidance motivation. Less research has been conducted to analyze how certain objective features or changes in the organizational environment, such as the organizational AI adoption, influence employees’ approach or avoidance motivation, and thereby affect their work behavior.

Thus, by applying this approach-avoidance framework, we suggest that approach and avoidance motivations may significantly influence how organizational AI adoption relates to employees’ approach and avoidance behaviors. Meanwhile, our research has expanded the application scope of this theory by explaining, from the perspective of approach-avoidance motivation, how employees voluntarily adjust their working behaviors to cope with external environmental changes.

### 2.2 The approach motivation pathway: AI-supported autonomy as the mediating mechanism

According to the approach-avoidance motivational theory, approach motivation is marked by positive emotionality, extraversion, and promotion-focused strategies (e.g., advancement, aspiration) (Elliot and Thrash, 2010). In contrast, avoidance motivation aligns with prevention focus and aversion to negative outcomes (Impett et al., 2014). Based on such a motivational mechanism, we propose that organizational AI adoption can promote AI-supported autonomy.

Artificial intelligence refers to computational systems possessing adaptive and reasoning abilities that enable them to undertake tasks traditionally requiring human intelligence (Oh and Ki, 2024). AI has sparked profound changes in workplace dynamics by leveraging advanced technologies and novel features, fundamentally altering the way organizations operate (Choi, 2021).

Organizational AI adoption refers to the behavior or management model in which a company invests in AI technology and actively introduces, learns, and adopts AI technology in order to gain a competitive advantage (Lin et al., 2024; Cheng et al., 2023). For example, companies in the service sector are progressively implementing AI-driven chatbots and virtual voice assistants to enhance the efficiency of their customer support processes (Ivanov et al., 2017). Embedding AI into organizational frameworks signifies a major technological evolution in contemporary work environments (Rampersad, 2020), a shift that is believed to heighten the demand for employees to effectively navigate and regulate their work responsibilities (Johnson et al., 2021).

Researchers in Human Resource Management (HRM) have widely studied factors influencing AI adoption at both organizational (Pan et al., 2022; Holm et al., 2023; Pillai et al., 2024) and individual levels (Chen et al., 2022; Pan, 2020; Sohn and Kwon, 2020). Increasing research also examines how AI integration affects HRM practices. Most studies focus on theoretical frameworks at industry and organizational levels, particularly how AI shapes labor markets and employment trends (Cramarenco et al., 2023; Patnaik and Bakkar, 2024). Within this area, a limited number of studies explore employees’ psychological and behavioral responses to AI from an individual perspective, mainly assessing its impact on job attitudes such as engagement (Nguyen and Vu, 2023), satisfaction (Prentice et al., 2023), and turnover intention (Li et al., 2021).

AI-supported autonomy reflects how AI enhances (rather than diminishes) employees’ perceived control over work methods, timing, and decisions (Wang et al., 2023) unlike traditional autonomy (Hackman and Oldham, 1975). It is a key work characteristic that promotes positive attitudes and behaviors (Humphrey et al., 2007). According to previous studies, AI-supported autonomy can enable employees to feel in control of their work, enhance happiness and satisfaction, and thereby increase trust in AI (Bai et al., 2025). Furthermore, the benefits perceived by employees from AI (such as autonomy) are consistent with the goals of approach orientation (Impett et al., 2014).

The application of AI in the workplace can bring many conveniences to employees in their work. For instance, they can have a detailed understanding of their job content through AI systems, and also use AI to formulate work plans and optimize work processes (Shen and Cui, 2024). These AIe technologies enable employees to independently manage their own work processes and progress without being restricted by time and space, and improve work quality and efficiency (Kong et al., 2023). This, in turn, fosters a sense of AI-supported autonomy, provides employees with a sense of wellbeing and satisfaction, and thereby forms a favorable impression of AI and trust in it.

Thus, we propose the following hypothesis:

*H1:* Organizational AI adoption is positively associated with employees’ AI-supported autonomy.

According to the approach-avoidance motivational theory (Elliot, 2006), approach motivation is marked by positive emotional tendencies and extroversion. It drives behaviors linked to the favorable elements of promotion-oriented strategies, including progress, growth, and ambition (Elliot and Thrash, 2010; Impett et al., 2014). This theory proposes that approach motivation involves triggering the activation or direction of behavior toward a favorable object or situation. In contrast, avoidance motivation inspire or guides behavior that is removed from an unfavorable one (Xu et al., 2021).

Approach job crafting behavior is defined as proactive, effortful, motivated, and goal-oriented actions aimed at enhancing work situations. This form of behavior focuses on achieving improvements by seeking additional resources, addressing hindering demands, or increasing challenging demands (Bruning and Campion, 2018; Zhang and Parker, 2019; Lu et al., 2022).

Based on the approach-avoidance motivational theory (Elliot, 2006), we suggest that when employees are activated by approach motivation, they have a higher tendency to proactively explore novel work methods and processes in response to AI adoption. Specifically, when organizations adopt AI, employees will experience the efficiency and autonomy that AI technology brings to their work processes through the use of AI to assist them (Meharunisa et al., 2024). This positive experience can activate employees’ approach motivation. Additionally, job autonomy, which allows employees to independently decide how, when, and in what order to perform tasks (Hackman and Oldham, 1975), is a key work characteristic that promotes positive attitudes and behaviors (Humphrey et al., 2007; Kong et al., 2024). If employees perceive the AI-supported autonomy, they may be more actively involved in job design (Hauptman et al., 2024). These employees may take the initiative to learn and explore AI functions in order to better integrate AI technology into their work processes. This engagement generates positive reactions toward AI-related job activities, which can manifest as approach job crafting behaviors (e.g., seeking resources, addressing hindering demands, or increasing challenging demands) (Bruning and Campion, 2018; Zhang and Parker, 2019). Therefore, when employees’ approach motivation is activated, they will exhibit proactive behaviors, such as approach job crafting.

Thus, we propose the following hypothesis:

*H2:* AI-supported autonomy is positively associated with employees’ approach job crafting behavior.

We then propose that AI-supported autonomy mediates the relationship between organizational AI adoption and employees’ approach job crafting behavior. Jobs that integrate AI adoption can help employees develop new skills, address gaps in their capabilities, and enhance both their motivation and efficiency (Chowdhury et al., 2023). For example, the combination of artificial intelligence and assistive technologies helps frontline staff better fulfill their work, thereby stimulating their interest and eagerness for their jobs, which can promote improved service hospitality in the long term (Qiu et al., 2022).

Furthermore, greater autonomy enhances flexibility and creates a more relaxed working environment, which motivates individuals to develop a proactive mindset and become more actively involved in AI-related learning and innovation. As a result, employees can proactively adjust their work practices and interactions with AI, redefining tasks or revising work processes (Kong et al., 2023).

Hence, we expect there exists a relationship between organizational AI adoption and employees’ approach job crafting behavior. Based on the approach-avoidance motivational theory (Elliot, 2006), organizational AI adoption may elicit individuals’ approach motivation (e.g., AI-supported autonomy) (Kong et al., 2024). Job autonomy will encourage employees’ approach job crafting behavior. Thus, we hypothesize as follows:

*H3:* AI-supported autonomy will mediate the relationship between organizational AI adoption and employees’ approach job crafting behavior.

### 2.3 The avoidance motivation pathway: AI anxiety as the mediating mechanism

Based on the approach-avoidance framework (Elliot, 2006), approach motivation refers to the activation of behavior that is directed toward positive stimuli, such as favorable objects, events, or possibilities. In contrast, avoidance motivation involves the activation of behavior aimed at moving away from negative stimuli, including undesirable objects, events, or scenarios (Elliot, 2006; Ferris et al., 2011, 2016).

In this study, we suggest that organizational AI adoption may also stimulate inclinations to avoid pressure and challenge (i.e., AI anxiety). AI anxiety refers to the concern or fear arising from the changes brought about by AI technologies in both individual lives and society at large (Kaya, 2021). AI anxiety represents a comprehensive emotional response characterized by anxiety or fear, which serves to inhibit individuals’ engagement with AI (Wang and Wang, 2022). AI anxiety encompasses AI learning anxiety, AI configuration anxiety, job replacement anxiety, and sociotechnical blindness (Wang et al., 2024).

When organizations start to adopt AI into their workflow, employees may regard it as external pressure, leading them to experience emotions such as fear, anxiety, and worry. These negative emotions can activate employees’ avoidance motivation. Specifically, the organizational AI adoption can cause anxiety among employees related to the learning of AI. When employees find AI skills difficult to acquire, they may worry about learning them quickly, increasing their AI learning anxiety (Feng et al., 2025). Moreover, the rapid development of AI has fostered a competitive environment, prompting employees to develop AI-related competencies to maintain an edge over their colleagues, which results in heightened AI learning anxiety (Konuk et al., 2023; Hu et al., 2025).

In addition, the organizational AI adoption may also stimulate concerns among employees regarding job replacement anxiety. As AI is increasingly popular, employees may also view AI as a potential job threat. Employees fear that AI will eventually substitute themselves (Zhu et al., 2025; Teng et al., 2024). Previous work has reported that organizational AI adoption, a key information source in the work environment, could generate pressure among employees, who are worried about being replaced (Cheng et al., 2023). Such potential risks can also trigger negative affect, like anxiety and fear, in AI users (Yu et al., 2023). In our case, organizational AI adoption is more prone to activate employees’ AI anxiety.

In summary, we propose the following hypothesis:

*H4:* Organizational AI adoption is positively associated with employees’ AI anxiety.

Based on the basic principles of approach-avoidance motivation theory (Elliot, 2006), individuals driven by approach motives tend to engage in positive actions. In contrast, those guided by avoidance motives are more prone to exhibit negative behaviors. Taking these motivations into account, we suggest that employees’ AI anxiety may foster avoidance job crafting behaviors as a means to mitigate perceived threat caused by AI integration.

Avoidance job crafting is a proactive coping strategy that includes two aspects: avoidance resource crafting, which involves disengaging mentally or emotionally from work to conserve resources, and avoidance demands crafting, which means withdrawing from undesirable tasks or situations (Zhang and Parker, 2019; Lu et al., 2022). Avoidance job crafting behavior can be viewed as a proactive and purposeful reaction to avoidance motivation, aiming to escape from pessimistic emotions (i.e., AI anxiety). On the one hand, relevant empirical evidence indicates that there is a positive correlation between AI anxiety and negative attitudes toward AI, and the correlation is often stronger than that with positive attitudes (Wang and Qiu, 2023; Barnes, 2024). This negative emotional response may activate the avoidance motivation. On the other hand, a large amount of literature related to job crafting also indicates that individuals may adopt avoidance job crafting behavior as a strategy to cope with adverse conditions and relieve related stress (Rudolph et al., 2017; Petrou et al., 2018). Therefore, when employees experience AI anxiety, they may develop negative emotions toward AI technology. Such negative emotions will activate employees’ avoidance motivation, leading to neither acceptance nor trust of it (Peng and Wan, 2024). When employees develop avoidance motivation, they may be reluctant to utilize AI for work-related tasks. It may also increase other negative behaviors, such as avoidance job crafting behaviors (Lu et al., 2022).

Consequently, AI anxiety, by generating negative emotions and activating avoidance motivation, may prompt employees to engage in avoidance job crafting behaviors.

To conclude, we propose the following hypothesis:

*H5:* AI anxiety is positively associated with employees’ avoidance job crafting behavior.

Combining Hypothesis 4 and Hypothesis 5, we propose that organizational AI adoption has a positive indirect effect on employees’ avoidance job crafting behavior through AI anxiety. According to the approach-avoidance motivational theory (Elliot, 2006), organizational AI adoption as an external situation factor will increase employees’ workload, and they will face more complex work demands, which may require a significant portion of their time and energy (Konuk et al., 2023). This might increase employees’ emotional anxiety as employees worry about their capacity for learning and applying AI-related knowledge in their work.

Besides, the rapid advancement of artificial intelligence has demonstrated superior performance in certain areas, raising employee concerns about job displacement. This perception of AI as a psychological threat can deplete psychological resources and trigger negative emotions, leading to AI anxiety (Zhang and Tong, 2024). This type of anxiety activates employees’ avoidance motivation, causing them to develop a negative attitude toward AI technology or products and exhibit avoidance behaviors, such as withdrawing from unfavorable situations or avoiding complex tasks. Eventually, it may prompt employees to engage in avoidance job crafting behaviors (such as avoidance resource crafting and avoidance demands crafting) as a coping strategy to deal with complex external situations and alleviate AI anxiety.

Thus, we propose the following hypothesis:

*H6:* AI anxiety mediates the relationship between organizational AI adoption and employees’ avoidance job crafting behavior.

### 2.4 AI knowledge sharing as the boundary condition

To further investigate when the employees of the organization who adopt AI are more likely to be activated in approach motivation (i.e., AI-supported autonomy) and less likely to undergo activation in avoidance motivation (i.e., AI anxiety), we examine the moderating effect of AI knowledge sharing. We propose that AI knowledge sharing could enhance the relationship between organizational AI adoption and AI-supported autonomy while mitigating the relationship between organizational AI adoption and AI anxiety.

Knowledge is a distinctive, socially complex and evolving organizational asset (Grant, 1996). Sharing of knowledge (knowledge sharing) implements a codification strategy, to enable knowledge to be widely deployed and reused in the organization (Hansen, 1999). As noted in the literature, knowledge sharing is an important process that supports effective creation, use and preservation of AI capabilities.

It is also regarded as a crucial factor in promoting the adoption of AI-related technologies in organizations (Upadhyay et al., 2022; Rulandari and Silalahi, 2025). Thus, AI knowledge sharing refers to organizations using knowledge management strategies to facilitate employee sharing of AI concepts, practical applications, use cases, and collaborative methods for creating and exchanging knowledge (Chowdhury et al., 2022).

According to approach-avoidance motivational theory (Elliot, 2006), the distinction between approach and avoidance represents a fundamental aspect of modern motivation theory, including achievement goals (Dweck, 1986), achievement motives (McClelland, 1985), and regulatory foci (Higgins, 1997). Achievement motives refer to the general tendency to pursue competence and avoid incompetence (McClelland, 1985). The need for achievement reflects a driving force of pride associated with success, while the fear of failure stems from the desire to avoid the embarrassment associated with failure (Sommet et al., 2019). Importantly, both of these motives are deeply rooted in the context of social comparison (Ryan, 1967). On the one hand, when AI knowledge is shared within an organization, the organization can create mechanisms for employees to learn and exchange information on AI technology. For instance, this can be achieved through seminars, employee training sessions, employee knowledge forums, online knowledge bases, and other forms of learning and exchange related to AI (Kazmaci et al., 2025). Due to knowledge sharing, employees recognize the potential advantages of using artificial intelligence to complete work tasks, including improving work efficiency and task performance (Wu et al., 2024), which activates their achievement motivation under the approach-avoidance theory. Employees hope to make more active use of AI technology and gain greater autonomy in their work activities (Bai et al., 2025).

Therefore, they will be more proactive in learning the usage methods of AI and actively make concerted efforts to work collaboratively with AI, thereby enhancing AI-supported autonomy.

Thus, we suggest that AI knowledge sharing will enhance the positive impact of organizational AI adoption on AI-supported autonomy.

On the other hand, when AI knowledge sharing occurs within an organization, internal knowledge sharing activities establish a mechanism and culture that facilitates the exchange and learning of AI knowledge among employees. According to the approach-avoidance motivational theory (Elliot, 2006), knowledge-sharing activities will activate the avoidance incompetence tendency in employees’ achievement motives (McClelland, 1985), which represents the fear of failure and a desire to avoid the shame associated with it (Atkinson, 1964; Birney et al., 1969). Employees want to use AI to support their work and are unwilling to let anxiety about learning and using AI reduce its effectiveness or harm their performance (Chang et al., 2024). In consequence, with the support of AI knowledge-sharing activities within the organization, employees will actively and proactively acquire AI knowledge, master AI work skills, improve work efficiency, enhance their sense of work efficacy, and thereby reduce AI anxiety.

Therefore, we propose that, as a positive external situation, AI knowledge sharing can buffer the impact of organizational AI adoption on employees’ AI anxiety.

Thus, we propose the following hypotheses:

*H7:* AI knowledge sharing moderates the impact of organizational AI adoption on AI-supported autonomy; the relationship is stronger when organizations have a high level of AI knowledge sharing.

*H8:* AI knowledge sharing moderates the impact of organizational AI adoption on AI anxiety; the relationship is weaker when organizations have a high level of AI knowledge sharing.

Building on the proposed mediation and moderation hypotheses, we propose a moderated mediation model. AI knowledge sharing moderates the relationship between organizational AI adoption and employees’ approach or avoidance motivation, subsequently influencing job crafting behaviors. Specifically, in organizations with high levels of AI knowledge sharing, the indirect effect of organizational AI adoption on approach job crafting behavior through AI-supported autonomy is stronger. In contrast, it is weaker for those with limited knowledge of AI. Conversely, for organizations with high-level AI knowledge sharing, the indirect effect of organizational AI adoption on avoidance job crafting behavior through AI anxiety is weaker, whereas it is stronger for those with low-level AI knowledge sharing. Thus, we hypothesize:

*H9:* AI knowledge sharing moderates the indirect effect of organizational AI adoption on employees’ approach job crafting behavior through their AI-supported autonomy, such that this indirect relationship is stronger for organizations with high levels of AI knowledge sharing and vice versa.

*H10:* AI knowledge sharing moderates the indirect effect of organizational AI adoption on employees’ avoidance job crafting behavior through their AI anxiety, such that this indirect relationship is weaker for organizations with high levels of AI knowledge sharing and vice versa.

**FIGURE 1:**
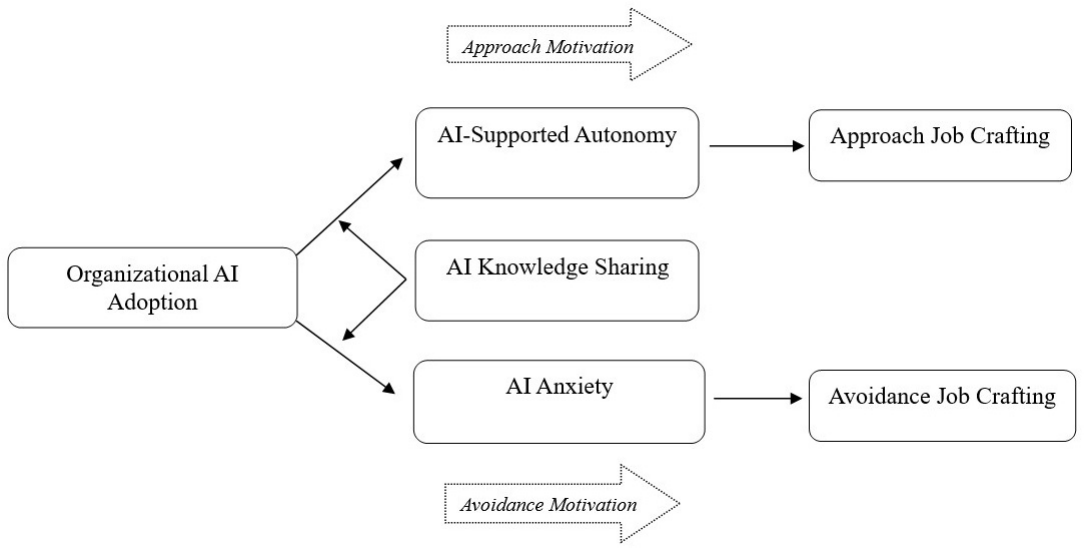
Proposed theoretical model.

Based on the proposed hypotheses above, the theoretical model of this study is illustrated as follows (see Figure 1).

## 3 Research method

### 3.1 Sample and procedure

This research utilized a comprehensive approach to data collection, focusing specifically on employees from diverse companies that utilize AI technologies or devices extensively, across various regions and industrial sectors. Participant selection was conducted using snowball sampling, a technique deemed suitable for the Chinese context (Sun et al., 2007), to reach diverse and hard-to-reach populations.

A total of 650 participants were enrolled in the study, selected from five different companies based in major cities across China, including Beijing, Shanghai, Chongqing, Guangzhou, and Shenzhen. The participants were selected from a wide variety of industrial sectors, including information technology, finance, real estate, manufacturing, and retail. This strategy was intended to ensure a broad and varied representation of professionals from different domains. Furthermore, to mitigate common method bias, following the recommendations of Podsakoff et al. (2003), the data collection process was designed as a three-wave survey, with an interval of 1 month between each wave. At Time 1, participants answered questions about organizational AI adoption and provided demographic information. At Time 2, data were collected regarding AI-supported autonomy and AI anxiety. At Time 3, information was collected on approach-avoidance job crafting and AI knowledge sharing.

To implement the survey distribution process, we collaborated with HR managers or directors from these organizations to administer the questionnaires. The HR department selected participants for the survey by randomly choosing employees across different departments. To protect the privacy of employees, data collection was conducted through an online platform,^1^ enabling participants to submit their responses directly to the research team. Furthermore, to ensure anonymity, each employee was assigned a distinct code to link multiple rounds of data, rather than using personally identifiable information.

We clarified the academic purpose of utilizing the survey data and assured participants that their responses would remain confidential. For each round of the survey, every participating employee received a compensation of 6 RMB (approximately USD 0.90)—the survey process took place from April to June 2025.

### 3.2 Participants

During the initial phase of collecting survey data, we distributed questionnaires to 650 employees, resulting in 589 valid responses, which corresponded to an effective response rate of 90.6%. In the second phase of data collection, which took place 1 month later, the same group of 589 participants from the initial round was administered the questionnaires once again. This time, we received 546 valid responses, reflecting an improved effective response rate of 92.7%. In the final stage of questionnaire collection, after an additional month, we administered surveys to the 546 employees who had taken part in both the initial and second rounds. This resulted in 487 valid responses, achieving an effective response rate of 89.2%. The decrease in the number of participants observed throughout various phases of data collection can be attributed to the absence of specific individuals within the surveyed companies. This absence may have resulted from factors such as illness, business trips, or other individual factors.

The final analytical sample comprised 487 participants. The descriptive statistics for the valid participants indicate that the sample consisted of 54.0% males and 45.0% females. In terms of age distribution, 3.2% of the participants were between 18 and 24 years old, 14.1% were aged 25–29, 25.7% were in the 30–34 age group, 23.2% were 35–39 years old, 21.1% were 40–45 years of age, and 12.1% were older than 45. Regarding educational background, 24.6% of the participants had a junior college degree, 49.8% possessed a bachelor’s degree, and 25.4% held a master’s degree or higher. Concerning tenure, 52.7% had worked in this organization for 1–5 years, 28.9% for 6–10 years, 9.85% for 11–15 years, 5.1% for 16–20 years, and 3.3% for more than 20 years.

### 3.3 Measures

To ensure that the measures were equivalent in both the Chinese and English versions of the questionnaire, we adhered to Brislin (1983) guidelines. The original questionnaire was first translated into Chinese. Following this, two bilingual experts in foreign languages conducted a back-translation from Chinese back to English. Through multiple rounds of forward and reverse translation, this cyclical process persisted until reaching an acceptable level of semantic consistency.

#### 3.3.1 Organizational AI adoption

At Time 1, A three-item scale adapted by Lin et al. (2024) and Cheng et al. (2023) from Wang et al. (2016) were employed to assess the adoption of organizational AI. A 5-point Likert scale, ranging from 1 (strongly disagree) to 5 (strongly agree), is used for the following items. A sample item is: “My company has been involved in the adoption of AI technology.” The Cronbach’s alpha was 0.84.

#### 3.3.2 AI-supported autonomy

At Time 2, we adopted a 6-item scale from Kong et al. (2024) to measure AI-supported autonomy. A 5-point Likert scale ranging from 1 (strongly disagree) to 5 (strongly agree) is used for the following items. A sample item is as follows: “AI technology makes me feel that my work-related decisions reflect what I truly want.” The Cronbach’s alpha was 0.90.

#### 3.3.3 AI Anxiety

At Time 2, we used the 21-item scale developed by Wang and Wang (2022) measured employees’ AI anxiety. A sample item was “I am afraid that an AI technique may replace humans.” All items were scored using a 5-point Likert scale, ranging from 1 (never) to 5 (very often). The Cronbach’s alpha for this scale was 0.97.

#### 3.3.4 AI knowledge sharing

At Time 3, we used the 9-item scale from Chowdhury et al. (2022) to measure the organization’s AI knowledge sharing. A sample item was “My organization provides means and mechanisms to employees to share knowledge for AI adoption.” Participants evaluated each item using a 5-point Likert scale, with responses varying from 1 (strongly disagree) to 5 (strongly agree), with a Cronbach’s alpha coefficient of 0.97.

#### 3.3.5 Approach-avoidance job crafting

At Time 3, we used the 30-item scale from Bruning and Campion (2018) to measure approach-avoidance job crafting. Among them, there are 23 items concerning the approach to job crafting; a sample item is as follows: “Expand my role by providing opinions on important issues.” Cronbach’s alpha was 0.97. Furthermore, seven items measured avoidance job crafting; a sample item is as follows: “Work in a way that allows me to avoid others at work.” All items were rated on a 5-point Likert scale, ranging from 1 (never) to 5 (very often). The Cronbach’s alpha was 0.92.

#### 3.3.6 Control variables

Drawing on findings from previous studies that have established links between gender, age, education, and tenure with job crafting behaviors (Belanche et al., 2019; Sohn and Kwon, 2020; Yi and Choi, 2023), this research systematically controls for these demographic variables.

### 3.4 Data analysis

In this research, descriptive statistical analyses, internal consistency evaluations of the scales, and inter-variable correlation assessments were performed using SPSS 21.0. Confirmatory factor analysis (CFA) was conducted using Mplus 7.0. Furthermore, to test the proposed hypotheses, latent moderated structural equation modeling (LMS) was applied using Mplus 7.0, enabling the calculation of robust estimates and confidence intervals (Cheung and Lau, 2017).

In the context of hypothesis testing, we analyzed both a mediation effect and a moderated mediation effect, which is characterized by first-stage moderation. We utilized 10,000 bootstraps and a confidence interval (CI) to estimate the corresponding effects.

## 4 Results

### 4.1 Confirmatory method variance and discriminant validity

A single-factor test proposed by Harman was performed to assess the possible existence of common method bias (Podsakoff et al., 2003). The findings revealed that the first factor accounted for 22.06% of the overall variance, a figure notably lower than the standard threshold of 50% typically considered acceptable. This indicates that common method bias was not a significant concern in the current study.

To evaluate the discriminant validity among all variables under investigation, a series of confirmatory factor analyses were conducted, with the resulting model fit indices displayed in Table 1.

**TABLE 1:** Model fit results for confirmatory factor analyses.

Model	χ^2^	df	χ^2^/df	RMSEA	CFI	TLI	SRMR
1. Six-factor model	4781.226	2,262	2.113	0.048	0.916	0.912	0.031
2. Five-factor model ^a^	4669.792	2,267	2.059	0.047	0.911	0.907	0.055
3. Four-factor model ^b^	6822.665	2,271	3.004	0.064	0.831	0.825	0.100
4. Three-factor model ^c^	6948.051	2,274	3.055	0.065	0.826	0.820	0.100
5. One-factor model ^d^	21021.193	2,277	9.231	0.130	0.302	0.281	0.242

*N* = 487;

*^a^*Combining AI-supported autonomy with approach job crafting;

*^b^*Combining AI-supported autonomy with approach job crafting, combining AI anxiety with avoidance job crafting;

*^c^*Combining AI-supported autonomy with approach job crafting, combining AI anxiety with avoidance job crafting, combining organizational AI adoption with AI knowledge sharing;

*^d^*Combining all variables into one factor.

As presented in Table 1, the hypothesized six-factor model, consisting of organizational AI adoption, AI-supported autonomy, AI anxiety, AI knowledge sharing, approach job crafting, avoidance job crafting (χ^2^ = 4781.226, df = 2,262, CFI = 0.916, TLI = 0.912, SRMR = 0.031, and RMSEA = 0.048), fits the data well (i.e., χ^2^/df < 3, CFI > 0.90, TLI > 0.90, SRMR < 0.08, and RMSEA < 0.08). Additionally, chi-square difference tests were conducted to compare the proposed model with all competing models, indicating that the proposed model demonstrates the best data fit. This supports the discriminant validity of the constructs, as the hypothesized model demonstrated superior fit compared to all alternative models.

### 4.2 Descriptive statistics and correlations

Table 2 presents the descriptive statistics and bivariate correlations between the variables under investigation. The results indicate a positive association between organizational AI adoption and AI-supported autonomy (*r* = 0.145, *p* < 0.01). Additionally, organizational AI adoption was found to have a positive association with AI anxiety (*r* = 0.345, *p* < 0.01). Moreover, the findings revealed a significant positive correlation between AI-supported autonomy and approach job crafting (*r* = 0.367, *p* < 0.01). In contrast, AI anxiety displayed a positive correlation with avoidance job crafting (*r* = 0.149, *p* < 0.01). These results provide initial support for Hypotheses 1, 2, 4, and 5.

**TABLE 2:** Means, standard deviations, and correlations between the study variables.

Variables	Mean	SD	1	2	3	4	5	6	7	8	9
1. Gender	1.46	0.499									
2. Age	36.02	6.752	–0.66								
3. Education	16.36	1.338	–0.119*	0.341**							
4. Tenure	5.70	5.184	–0.036	0.460**	0.078						
5. Organizational AI adoption	3.555	1.156	0.021	–0.030	–0.047	–0.050					
6. AI-supported autonomy	3.544	0.906	0.010	–0.066	–0.083	–0.042	0.145**				
7. AI anxiety	3.241	0.878	–0.029	–0.021	0.055	–0.049	0.345**	0.033			
8. AI knowledge sharing	3.276	1.163	0.009	–0.008	–0.107*	–0.014	0.809**	0.108*	0.292**		
9. Approach job crafting	3.305	0.896	0.034	–0.029	–0.023	0.027	0.020	0.367**	–0.105*	0.025	
10. Avoidance job crafting	3.081	0.911	0.045	0.033	–0.053	0.051	0.060	–0.037	0.149**	0.074	0.376**

*n* = 487; *p < 0.05; **p < 0.01.

### 4.3 Hypothesis testing

This research employed Latent Moderated Structural (LMS) modeling, as implemented in Mplus 7.0, to assess the hypotheses, aiming to obtain robust estimates and confidence intervals (Cheung and Lau, 2017). Bootstrapping with 10,000 resamples was employed to assess the significance of indirect effects (Hayes, 2022).

Regarding Hypotheses 1 and 2, which posited a positive relationship between organizational AI adoption and AI-supported autonomy and a positive relationship between AI-supported autonomy and approach job crafting behavior. As shown in Table 3 (M1, M2), the statistical analysis demonstrated a significantly positive association between organizational AI adoption and AI-supported autonomy (β = 0.119, *p* < 0.001), as well as between AI-supported autonomy and approach job crafting (β = 0.37, *p* < 0.001). Consequently, Hypotheses 1 and 2 were supported.

**TABLE 3:** Unstandardized coefficient and standard error of moderated mediation path analysis.

Independent variables	Simple mediation				Moderated mediation	
	M1 (AI-supported autonomy) b (SE)	M2 (Approach job crafting) b (SE)	M3 (AI anxiety) b (SE)	M4 (Avoidance job crafting) b (SE)	M5 (AI-supported autonomy) b (SE)	M6 (AI anxiety) b (SE)
**Controls**						
Gender	–0.002(0.003)	0.000(0.002)	0.00 (0.002)	0.00 (0.002)	0.017 (0.080)	–0.063 (0.075)
Age	–0.002(0.003)	0.000(0.002)	0.00 (0.002)	0.00 (0.002)	–0.005 (0.005)	–0.001 (0.005)
Education	–0.002(0.003)	0.000(0.002)	0.00 (0.002)	0.00 (0.002)	–0.073 (0.053)	0.079 (0.052)
Tenure	–0.002(0.003)	0.000(0.002)	0.00 (0.002)	0.00 (0.002)	–0.001 (0.005)	0.000 (0.006)
Organizational AI adoption	0.119***(0.036)		0.263***(0.034)		0.233**(0.074)	0.168**(0.056)
AI-supported autonomy		0.370***(0.039)				
AI anxiety				0.151**(0.055)		
AI knowledge sharing					–0.056 (0.066)	0.051 (0.054)
OAA × AKS					0.164***(0.032)	–0.114***(0.027)
R2			0.121***(0.029)	0.022 (0.015)	0.167*(0.070)	0.103***(0.028)

*N* = 487. Bootstrap sample size = 10,000. *p < 0.05; ** *p* < 0.01; *** *p* < 0.001. OAA, Organizational AI adoption; AKS, AI knowledge sharing.

Hypotheses 4 and 5 predicted a positive relationship between organizational AI adoption and AI anxiety, as well as a positive relationship between AI anxiety and avoidance job crafting. Hypotheses 4 and 5 proposed that there would be a positive association between organizational AI adoption and AI anxiety, as well as a positive relationship between AI anxiety and avoidance job crafting. Statistical results (Table 3, M3, M4) revealed a significant positive relationship between organizational AI adoption and AI anxiety (β = 0.263, *p* < 0.001), as well as a significant positive relationship between AI anxiety and avoidance job crafting (β = 0.151, *p* < 0.01). Therefore, Hypotheses 4 and 5 were supported.

Hypotheses 3 and 6 proposed that AI-supported autonomy mediates the relationship between organizational AI adoption and approach job crafting. In contrast, AI anxiety serves as a mediating factor in the relationship between organizational AI adoption and employees’ avoidance job crafting. To investigate these mediating effects, we followed the methodology outlined by Hayes (2022). After accounting for the influence of AI-supported autonomy, the impact of organizational AI adoption on approach job crafting remained statistically significant (β = 0.044, *p* < 0.01), suggesting a pattern of partial mediation (Table 4, Simple mediation effect). Consistently, even after accounting for AI anxiety, the influence of organizational AI adoption on avoidance job crafting remained statistically significant (β = 0.040, *p* < 0.05), suggesting a partially mediating role (see Table 4, Simple mediation effect). Furthermore, the bootstrapping analysis confirmed the significance of the indirect effects [95% CI (0.019, 0.078); 95% CI (0.011, 0.076)], respectively. These findings provide support for Hypotheses 3 and 6.

**TABLE 4:** Simple mediation effect and moderated mediation effect.

		Indirect effect	SE	95%CI
**Simple mediation effect**				
AI-supported autonomy		0.044	0.015	[0.019 to 0.078]
AI anxiety		0.040	0.016	[0.011 to 0.076]
**Moderated mediation effect**				
AI-supported autonomy	High level of moderator	0.156	0.040	[0.087 to 0.241]
	Low level of moderator	0.015	0.024	[–0.032 to 0.061]
AI anxiety	High level of moderator	0.005	0.012	[–0.012 to 0.037]
	Low level of moderator	0.045	0.018	[0.014 to 0.089]

**FIGURE 2:**
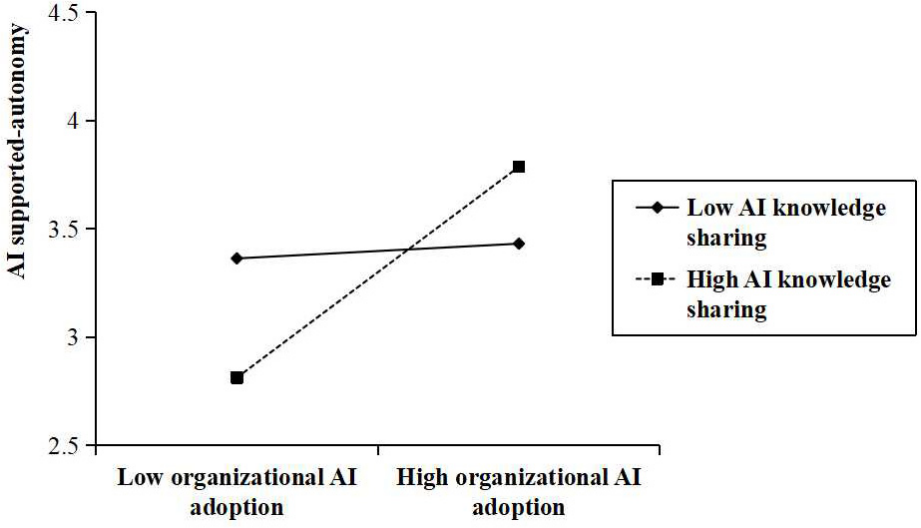
Moderating effect of AI knowledge sharing on the relationship between organizational AI adoption and AI-supported autonomy.

**FIGURE 3:**
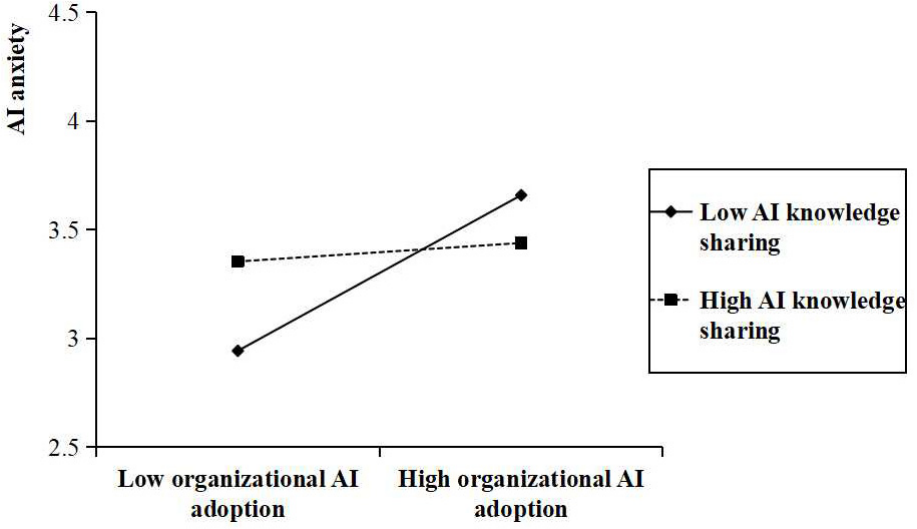
Moderating effect of AI knowledge sharing on the relationship between organizational AI adoption and AI anxiety.

Hypotheses 7 and 8 proposed that AI knowledge sharing plays a moderating role in the relationships between organizational AI adoption and both AI-supported autonomy and AI anxiety. As presented in Table 3, a statistically significant interaction was found between organizational AI adoption and AI knowledge sharing in influencing AI-supported autonomy (β = 0.164, *p* < 0.001). Similarly, a significant interaction was found between organizational AI adoption and AI knowledge sharing in predicting AI anxiety (β = –0.114, *p* < 0.001). Figures 2, 3 present interaction plots derived from values at one standard deviation above and below the mean of AI knowledge sharing. As shown in Figure 2, a positive correlation exists between organizational AI adoption and AI-supported autonomy, which is particularly evident within organizations with high levels of AI knowledge sharing than those with low levels of AI knowledge sharing. In contrast, Figure 3 illustrates that the positive relationship between organizational AI adoption and AI anxiety is weaker at higher levels of AI knowledge sharing than at lower levels. Consequently, Hypotheses 7 and 8 were supported.

Regarding moderated mediation (Hypotheses 9 and 10), Table 4 presents the relevant indices. The indirect effect of organizational AI adoption on approach job crafting through AI-supported autonomy varied significantly across various degrees of AI knowledge sharing. Specifically, the indirect impact was significantly positive when respondents perceived that the degree of AI knowledge sharing within the organization was high [β = 0.156, SE = 0.040, 95% biased-corrected CI (0.087, 0.241)]. At the same time, it was not significant when the degree of AI knowledge sharing was one SD below the mean (–1 SD) [β = 0.015, SE = 0.024, 95% biased corrected CI (–0.032, 0.061)]. This supports Hypothesis 9.

At the same time, the indirect impact of organizational AI adoption on avoidance job crafting through AI anxiety varied significantly across different levels of AI knowledge sharing. Specifically, the indirect effect was significantly positive when participants perceived that the degree of AI knowledge sharing within the organization was low [β = 0.045, SE = 0.018, 95% biased-corrected CI (0.014, 0.089)]. At the same time, it was not significant when the degree of AI knowledge sharing was one SD above the mean (+1 SD) [β = 0.005, SE = 0.012, 95% biased corrected CI (–0.012, 0.037)]. This lends support to Hypothesis 10.

## 5 Discussion

By incorporating the approach-avoidance motivational theory (Elliot, 2006), this research examines the mechanisms and conditions under which organizational AI adoption affects employees’ approach-avoidance job crafting behaviors. This study yields two significant research findings. First, empirical analysis shows that organizational AI adoption improves employees’ approach job crafting by activating AI-supported autonomy, while also stimulating avoidance job crafting behavior by eliciting AI anxiety. It indicated that employees’ approach or avoidance motivation regarding organizational AI adoption is crucial for their job crafting behavior. Moreover, this study validated the role of AI knowledge sharing in moderating the indirect effects that organizational AI adoption has on approach or avoidance job crafting behaviors via approach motivation (i.e., AI-supported autonomy) or avoidance motivation (i.e., AI anxiety). The findings indicate that employees who develop greater knowledge, skills, or capabilities in utilizing AI technology are more likely to handle stress and uncertainty, and consequently, craft their jobs.

### 5.1 Theoretical implications

This study offers the following three theoretical contributions.

Firstly, this research contributes to the existing research on the application of AI technology by introducing organizational AI adoption as an antecedent factor and revealing its relationship with job crafting behaviors. Prior AI related studies on enterprise management concentrated on analyzing from one employee view, such as the AI adoption (Pan, 2020; Bakir et al., 2025) or AI awareness (Kong et al., 2024; Wang et al., 2022) on employee behavior from the perspective of individual employees. We find that insufficient attention has been devoted to studying organizational AI adoption as an external environmental factor and its psychological and behavioral impacts on employees. This research fills the existing gap by examining the dual impact of organizational AI adoption, with a particular focus on its influence on approach-avoidance job crafting behaviors. It thereby can build a meaningful basis of understanding what employees may do with a job crafting approach in AI environments.

Secondly, based on the approach-avoidance motivational theory (Elliot, 2006), this research uncovers the underlying psychological processes by which organizational AI adoption affects employees’ approach or avoidance job crafting. It does so by identifying approach motivation (i.e., AI-supported autonomy) and avoidance motivation (i.e., AI anxiety) as key mediating factors. The results suggest that organizational AI adoption affects employees’ job crafting through different motivational pathways. This finding provides new insights into the complex relationship between organizational technological change and employee behavior. Besides, extends the approach-avoidance motivational theory to the application scenarios of organizational AI adoption, expanding and supplementing the scope of application of the existing theoretical framework.

Thirdly, this study contributes to a deeper comprehension of the significance of knowledge management within organizational contexts by exploring the moderating effects of AI knowledge sharing. Particularly, when the level of AI knowledge sharing within an organization is high, organizational AI adoption leads to a reduction in AI anxiety and avoidance job crafting behavior; at the same time, it promotes AI-supported autonomy and increases approach job crafting behavior. Conversely, for organizations with low AI knowledge sharing, organizational AI adoption leads to reduced AI-supported autonomy and diminished approach job crafting behavior; meanwhile, it also leads to increased AI anxiety and greater avoidance job crafting behavior. This also provides valuable insights for relevant research on AI-related knowledge management strategies within the organization.

### 5.2 Practical implications

Our research shows that organizational AI adoption activates diverse motivational responses in employees, which in turn prompt them to engage in either approach or avoidance job crafting.

Managers should recognize the significance of organizational AI adoption and its dual impact on employees’ job crafting behaviors. Furthermore, during this process, an effective organizational support system is crucial (Duke et al., 2009). Therefore, managers need to make every effort to construct an aid supportive environment in order to encourage workers to increase autonomy and thus realize this kind of approach job crafting. Specifically, by providing clear explanations and consistent feedback, organizations can improve employees’ understanding and engagement with AI systems, thereby strengthening their sense of control and autonomy (Yu et al., 2023). Additionally, organizations can leverage real case studies of successful AI applications to demonstrate the tangible benefits of AI integration, enabling employees to experience these advantages firsthand.

Given that the adoption of AI within organizations is an inevitable trend in the present era, managers should plan to mitigate the potential negative impacts on employees. Enterprises can offer psychological guidance and mentoring programs to help employees adapt to changes in job processes and maintain their mental health (Wei and Li, 2022), thereby reducing AI anxiety. Moreover, the enterprises also needs to give confidence to employees’ careers and let the employees know that the role of AI is assisting them in their productivity enhancement and not substituting their job roles, which would alleviate employees’ concerns (Zhao et al., 2025). Moreover, organizations can provide career planning consultations and job rotation programs to help employees develop AI-related career paths and gain practical experience applying AI in their work, reducing or alleviating AI anxiety.

In addition, companies should emphasize AI knowledge sharing by implementing organized internal communication systems and support mechanisms, thereby fostering a collaborative environment (Kong et al., 2023). By means of learning events, internal training and involvement in AI groups, nurturing a culture of knowledge sharing and role-modeling encouraging employees to involved more in AI, resulting in a higher approach motivation (such as AI-supported autonomy), and less avoidance motivation (such as AI anxiety). Besides, organizations should focus on improving employees’ ability to collaborate with AI, such as enhancing data literacy and promoting the responsible use of AI, in order to utilize AI more effectively and ethically, and reduce negative attitudes toward AI adoption.

### 5.3 Limitations and future research

Some limitations of this study warrant discussion, along with their implications for future research.

Although we employed a three-wave data collection process and addressed common method variance using Harman’s single-factor test, our reliance on self-reported measures remains a limitation. Future research can strengthen methodological rigor by incorporating multi-source data, such as supervisor ratings of employees’ AI adoption behaviors and peer assessments of knowledge-sharing activities. Current measures of approach/avoidance job crafting still lack AI-specific content, reflecting the nascent stage of research in this area (Li et al., 2024). Key questions remain about how employees adapt their jobs to AI, how effective these adaptations are, and what factors influence such behaviors differently. Future work can create and verify AI-specific job crafting scale, identify the antecedents and consequences of crafting behavior in relation to AI, examine the contextual factors affecting crafting behavior in AI-influenced work settings.

## 6 Conclusion

This research investigates the mechanisms and conditions under which organizational AI adoption affects employees’ job crafting behavior. The findings indicate that the organizational AI adoption triggers approach or avoidance motivational reactions, thereby influencing employees’ job crafting behavior. Specifically, AI-supported autonomy mediates the positive relationship between organizational AI adoption and approach job crafting. In contrast, AI anxiety mediates the positive relationship between organizational AI adoption and avoidance job crafting. Moreover, AI knowledge sharing moderates the impact of organizational AI adoption on motivational responses. High levels of AI knowledge sharing strengthen the relationship between organizational AI adoption and AI-supported autonomy, while weakening the relationship between organizational AI adoption and AI anxiety, thereby shaping subsequent job crafting.

## Data Availability

The raw data supporting the conclusions of this article will be made available by the authors, without undue reservation.

## Footnotes

1
https://www.wjx.cn
